# Refractory *Helicobacter pylori* infection and the gastric microbiota

**DOI:** 10.3389/fcimb.2022.976710

**Published:** 2022-09-27

**Authors:** Dongsheng Liu, Jinyun Wang, Yong Xie

**Affiliations:** ^1^ Digestive Disease Hospital, The First Affiliated Hospital of Nanchang University, Nanchang, China; ^2^ Department of Gastroenterology, The First Affiliated Hospital of Nanchang University, Nanchang, China; ^3^ JiangXi Clinical Research Center for Gastroenterology, The First Affiliated Hospital of Nanchang University, Nanchang, China

**Keywords:** Refractory *H. pylori* infection, *H. pylori* eradication, gastric microbiota, 16S rRNA sequencing, bacteria functional prediction

## Abstract

**Background:**

Curing refractory *Helicobacter pylori* infection is difficult. In addition, there is currently no research on the gastric microbiota of refractory *H. pylori* infection.

**Methods:**

We designed a clinical retrospective study involving 32 subjects divided into three groups: 1. nAGHp.a, treatment-naïve patients with *H. pylori* infection; 2. nAGHp.b, *H. pylori*-negative patients; and 3. EFHp.a, patients with refractory *H. pylori* infection. Gastric mucosal samples from the biobank of our research center were collected for 16S rRNA sequencing analysis and bacterial functions were predicted *via* PICRUSt.

**Results:**

There were significant differences between the *H. pylori-* positive group and the *H. pylori-*negative group in species diversity, gastric microbiota structure, and bacterial function. The beneficial *Lactobacillus* in the *H. pylori*-positive group were significantly enriched compared with those in the refractory *H. pylori* infection group. The bacterial interaction network diagram suggested that the microbiota interactions in the refractory *H. pylori* infection group decreased. The gastric microbiota of the refractory *H. pylori* infection group was enriched in the pathways of metabolism and infectious diseases (energy metabolism, bacterial secretion system, glutathione metabolism, protein folding and associated processing, sulphur metabolism, membrane and intracellular structural molecules, lipopolysaccharide biosynthesis, ubiquinone and other terpenoid-quinone biosynthesis, inorganic ion transport and metabolism, and metabolism of cofactors and vitamins) when compared with the *H. pylori*-positive group without treatment based on PICRUSt analysis.

**Conclusion:**

Significant alterations occurred in the gastric microbiota when eradication of *H. pylori* failed multiple times. A history of eradication of multiple *H. pylori* infections leads to an imbalance in the gastric mucosal microbiota to a certain extent, which was mainly reflected in the inhibition of the growth of beneficial *Lactobacillus* in the stomach. Patients with refractory *H. pylori* infection may be at a higher risk of developing gastric cancer than other *H. pylori*-positive patients.

## Introduction

Since Marshall and Warren discovered *Helicobacter pylori* in 1983 (Marshall and Warren, 1984), the international community focused on digestive research has made breakthrough progress in the etiology and treatment of chronic gastritis, peptic ulcer disease, and gastric cancer. Over 50% of people are infected with *H. pylori*, 15–20% of *H. pylori* -infected people can develop gastric peptic ulcer disease, and the incidence of gastric cancer in *H. pylori* -infected people is 2–6 times higher than that in noninfected people (Yu et al., 2017). At present, the World Health Organization (WHO) regards *H. pylori* as a class I carcinogen of gastric cancer (Wang et al., 2014). Treatment of *H. pylori* infection has been the main strategy for preventing gastric cancer (Choi et al., 2018).

In recent years, with the application of high-throughput sequencing technology, progress has been made in understanding gastric microbiota. Many previous studies focused mainly on the role of changes in the gastric microbiota in the occurrence and development of gastric diseases as well as the association between the gastric microbiota and *H. pylori* infection (Zhang et al., 2005; Aviles-Jimenez et al., 2014; Eun et al., 2014; Coker et al., 2018; Dai et al., 2021; Niu et al., 2021; Yuan et al., 2021; Wu et al., 2022). However, no study has evaluated the gastric microbiota in refractory *H. pylori* infection. Because of increasing antimicrobial resistance, curing *H. pylori* infection is difficult, and eradication rates are often <70% (Malfertheiner et al., 2017). Eradication of *H. pylori* infection fails in many subjects multiple times, and eradication can still be unsuccessful after multiple treatments with different antibiotics. Therefore, related gastric microecology research is particularly important.

In this study, 16S rRNA amplicon sequencing technology was used to analyze the gastric microbiota of treatment-naïve patients with *H. pylori* infection, patients without *H. pylori* infection, and patients with refractory *H. pylori* infection, to clarify the characteristics of the gastric microbiota in refractory *H. pylori* infection and provide a new theoretical basis for treating refractory *H. pylori* infection in the future.

## Methods

### Patients and samples

The gastric mucosal samples included in this study were derived from the biological specimen bank of our research center. Patients who underwent gastroduodenoscopy were identified in 2019 at the First Affiliated Hospital of Nanchang University. The inclusion criteria were as follows: (a) patients who were 18-70 years old; (b) patients who had not used antibiotics, probiotics, proton pump inhibitors(PPIs), synbiotics, bismuth, hormones, or immunosuppressants in the past 6 months; and (c) patients with gastroduodenoscopy results, indicating that the gastric mucosa was normal. Antrum biopsy samples were collected for microbiota analysis. *H. pylori* infection was tested by using the rapid urease test (RUT). This study divided the patients into three groups according to *H. pylori* status and therapy as follows: group nAGHp.a: treatment-naïve patients with *H. pylori* infection; group nAGHp.b (control group): *H. pylori*-negative patients; and group EFHp.a: patients with refractory *H. pylori* infection. We followed up with the group of nAGHp.a patients to confirm their first successful eradication of *H. pylori.* Specific information on previous *H. pylori* eradication drugs in the EFHp.a group was collected (**Table S1**).

This study was approved by the Ethics Committee of the First Affiliated Hospital of Nanchang University (2018-019-1), and informed consent was obtained from participants before the endoscopy.

### Relevant definitions

Refractory *H. pylori* infection is defined by a persistently positive non-serologic *H. pylori* test result (ie, a breath-, stool-, or gastroscopy-based test), at least 4 weeks after 1 or more completed course(s) of a current guideline-recommended first-line *H. pylori* eradication therapy, and off of any medications, such as PPIs, that might impact the test sensitivity (Shah et al., 2021).

### Bacterial genomic DNA extraction

Gastric mucosal samples were collected immediately into a sterile tube, transported to the laboratory within 15 minutes on ice, and stored at −80°C until use. DNA was extracted by using a tissue DNA kit (D3396; Omega), and a physical disruption to break all types of bacteria by glass sand grinding in heated cetyltrimethylammonium bromide solution was included. The total DNA was eluted in 50 μL of elution buffer and stored at −20°C.

### Amplification and sequencing of 16S rRNA

The V4 region of the 16S rRNA gene was amplified by using universal primers 515F/806R to construct a 16S amplicon library. The library was constructed using a TruSeq ^®^ DNA PCR-Free Sample Preparation Kit(Illumina, USA). The constructed library was quantified by Qubit(Invitrogen) and Q-PCR. The library was qualified and sequenced on a NovaSeq 6000.

### Bioinformatics analysis

Original data were analyzed by bioinformatics under the default parameters of QIIME software (Version 1.9.1). Sequences with ≥97% similarity were assigned to the same operational taxonomic units (OTUs). Alpha diversity (Chao1, Shannon) was calculated, and beta diversity was presented by weighted UniFrac of principle coordinate analysis (PCoA). The different bacterial compositions among groups were analyzed by the Metastats algorithm. To identify biomarkers with differentiating abundance in the different groups, the linear discriminant analysis (LDA) effect size (LEfSe) algorithm was used. LEfSe couples robust tests for measuring statistical significance (Kruskal-Wallis test) with quantitative tests for biological consistency (Wilcoxon rank sum test). Based on species abundance, the correlation coefficient (Spearman correlation coefficient) between each genus was calculated, and the correlation coefficient matrix was obtained. Cytoscape V.3.6.1 was used for the visualization of networks with significant correlations between genera.

### Metagenomics by PICRUSt

The functionality of the different metagenomes grouped by sample type was predicted using the software PICRUSt. PICRUSt was used to derive relative Kyoto Encyclopedia of Genes and Genomes (KEGG) pathway abundance.

### Statistical analysis

SPSS 26 was used for statistical analysis. The T test or Wilcoxon rank sum test was used for comparison between the two groups. One-way ANOVA was used for comparisons among the three groups. FDR analysis for KEGG pathways was used to correct *P* values. All statistical tests performed were two-tailed, and *P* values > 0.05 were considered nonsignificant, *P* values ≤ 0.05 = *, and *P* values ≤ 0.01 = **.

## Results

### Patient characteristics

In this study, 32 patients were enrolled, and 32 samples were collected. Group nAGHp.a comprised 12 patients, group nAGHp.b comprised 12 patients, and group EFHp.a comprised eight patients. The clinical information of the patients is presented in **Table 1**. There was no significant difference in age, sex, or BMI among the three groups. The specific information of the eight patients with refractory *H. pylori* infection is shown in **Table S1**.

**Table 1:** Clinical baseline data among three groups.

	nAGHp.a	nAGHp.b	EFHp.a	*P* value
Number Age (year)	12 46.67 ± 14.18	12 46.5 ± 15.81	8 49.5 ± 8.67	0.678
Sex (female,%)	6 (50%)	4 (33.33%)	49 (50%)	0.874
BMI (Kg/m^2^)	21.58 ± 1.67	21.49 ± 1.90	22.38 ± 1.76	0.588

BMI, body mass index; nAGHp.a, treatment-naïve patients with H. pylori infection; nAGHp.b, H. pylori-negative patients; EFHp.a, patients with refractory H. pylori infection.

### Rarefaction curves

**Figure 1:**
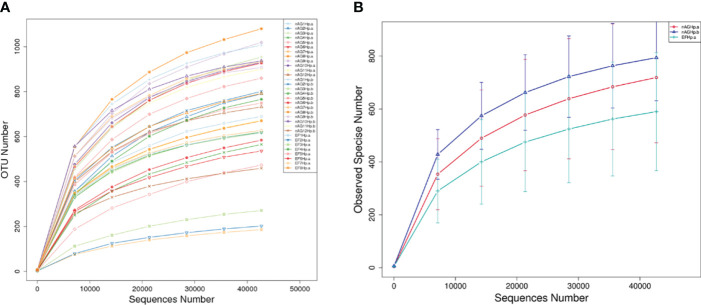
Rarefaction curves based on independent samples **(A)** and groups **(B)**. nAGHp.a, treatment-naïve patients with *H*. *pylori* infection; nAGHp.b, *H*. *pylori*-negative patients; EFHp.a, patients with refractory *H*. *pylori* infection.

With increasing sequencing depth, the dilution curves of each sample gradually reached the platform stage (**Figure 1A**), indicating that the current sequencing data were sufficient to cover most species and met the requirements for subsequent analysis. **Figure 1B** shows that the number of species observed from most to least was group nAGHp.b > group nAGHp.a > group EFHp.a.

### Alpha diversity

**Figure 2:**
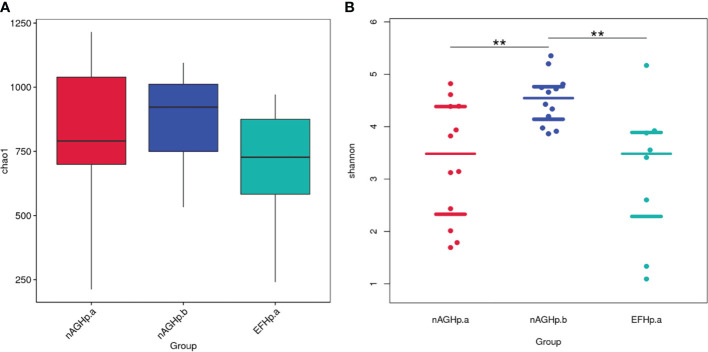
Comparisons of bacterial diversity index among groups. Chao1 index **(A)**, Shannon index **(B)**. **P < 0.01. nAGHp.a, treatment-naïve patients with *H. pylori* infection; nAGHp.b, *H. pylori*-negative patients; EFHp.a, patients with refractory *H. pylori* infection.

Alpha diversity reflects community richness and diversity and was presented by the Chao1 estimator and Shannon index, respectively. After leveling the platform sequencing data, the alpha diversity index was statistically analyzed. The alpha diversity index values were lower in the *H. pylori*-positive group than *H. pylori*-negative group (*P* < 0.01). However, among the *H. pylori*-positive patients, there was no difference in community richness and diversity between patients with or without eradication treatment (*P >*0.05). These results are shown in **Figure 2**.

### Beta diversity

**Figure 3:**
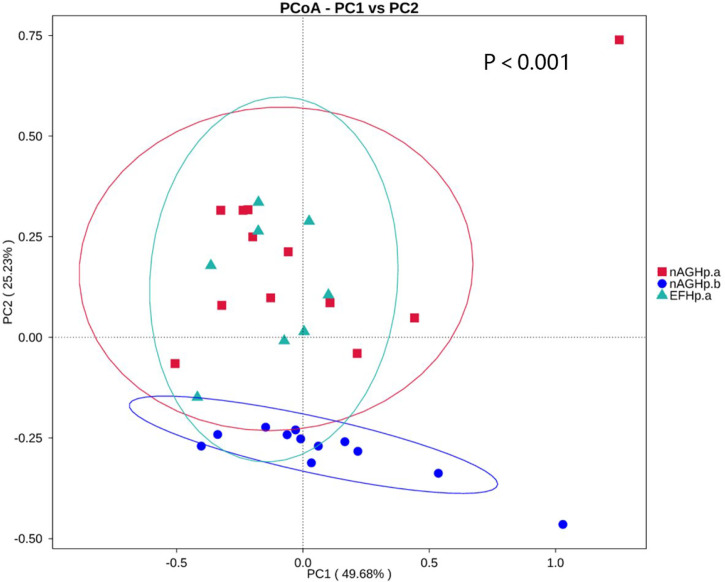
PCoA based on weighted UniFrac distance of the gastric microbiota among groups. nAGHp.a, treatment-naïve patients with *H. pylori* infection; nAGHp.b, *H. pylori*-negative patients; EFHp.a, patients with refractory *H*. *pylori* infection.

Beta diversity presents similarities in community structure and was analyzed using PCoA based on weighted UniFrac distance by bacterial abundance clustering. A significant similarity difference was found between the *H. pylori*-positive group and the *H. pylori*-negative group. In addition, the distances demonstrated that there were high similarities between group EFHp.a and group nAGHp.a. The PCoA results of weighted UniFrac distances are shown in **Figure 3**.

### Species composition and relative abundance

**Figure 4:**
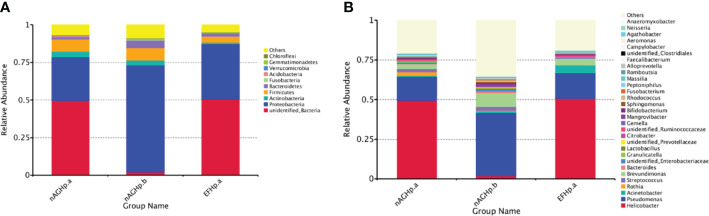
Relative abundance of gastric microbiota at phylum **(A)** level and genera **(B)** level among groups. nAGHp.a, treatment-naïve patients with *H*. *pylori* infection; nAGHp.b, *H*. *pylori*-negative patients; EFHp.a, patients with refractory *H*. *pylori* infection.

The column diagram of the relative abundance of species preliminarily indicated that at the phylum level, the proportion of the relative abundance of *Firmicutes* and *Actinobacteria* in the EFHp. a group was lower than that in the nAGHp. a group, while that of *Proteobacteria* and *Bacteroidetes* was higher than that in the nAGHp. a group (**Figure 4A**). At the genus level, the relative abundance of *Acinetobacter* in the EFHp.a group was higher than that in the nAGHp.a group, and the relative abundance of *Rothia* was lower (**Figure 4B**).

**Figure 5:**
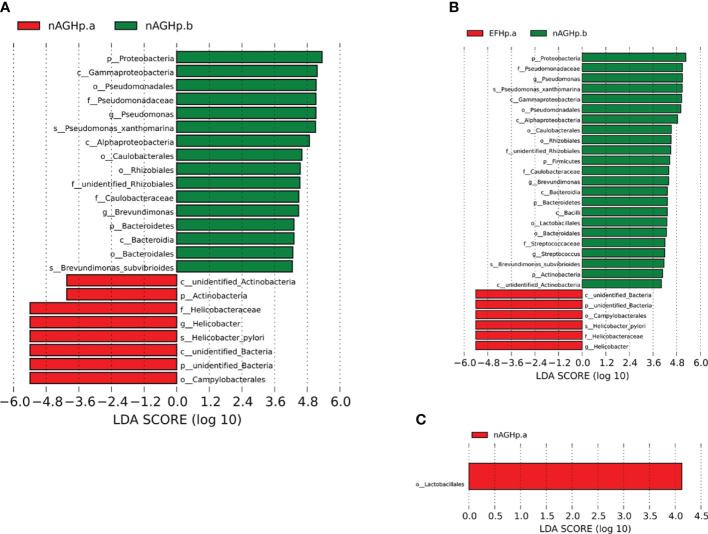
Identification of differential bacteria by LEfSe analysis. **(A)** Group nAGHp.a VS. Group nAGHp.b. **(B)** Group EFHp.a VS. Group nAGHp.b. **(C)** Group nAGHp.a VS. Group EFHp.a. nAGHp.a, treatment-naïve patients with *H*. *pylori* infection; nAGHp.b, *H*. *pylori*-negative patients; EFHp.a, patients with refractory *H. pylori* infection.

The biomarkers (the key bacterial members) among the three groups were further screened by using LEfSe. The LDA scores showed that *Actinobacteria* were enriched in the nAGHp.a group compared with the *H. pylori*-negative group at the phylum level **(** **Figure 5A** **)**. *Actinobacteria*, *Firmicutes*, *Bacteroidetes*, and *Proteobacteria* were enriched in the *H. pylori*-negative group compared with the EFHp.a group at the phylum level (**Figure 5B**). At the order level, *Lactobacillales* were enriched in group nAGHp.a compared with group EFHp.a (**Figure 5C**). Because the LDA threshold settings were too high, many genera were not displayed. We tried to obtain more differences at the genus level by further drawing a heatmap of species differences among these groups. Indeed, we found that the relative abundance of *Lactobacillus* belonging to *Lactobacillales* was significantly lower in the EFHp.a group than in the nAGHp.a group (**Figure S1**).

### Interaction network diagram of the microbiota

**Figure 6:**
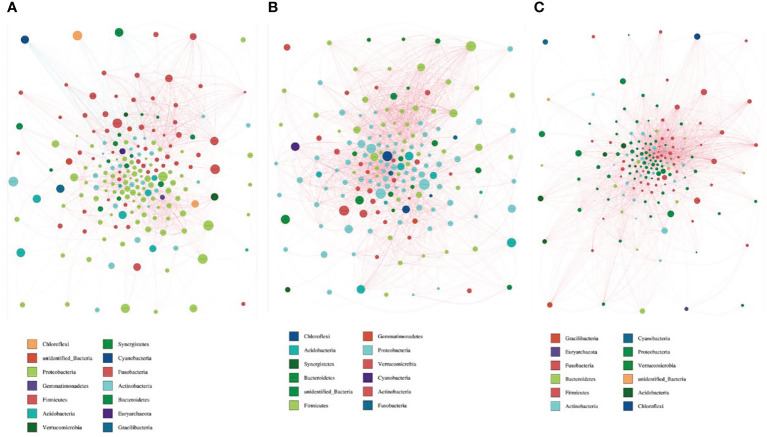
Bacterial interaction network diagram of nAGHp.b **(A)**, nAGHp.a **(B)**, EFHp.a **(C)**. Different nodes represent different genera, and node size represents the average relative abundance of the genus. The node color of the same door is the same genera. The thickness of the connection between nodes is positively correlated with the absolute value of the correlation coefficient of species interaction; the red line represents a positive correlation, and the blue line represents a negative correlation).

By drawing the microbiota interaction network diagram, we found that there were 14 species interacting with each other in group nAGHp.b **(** **Figure 6A** **)**, and 12 species in group nAGHp.a (**Figure 6B**). The microbiota interaction in the *H. pylori*-positive group was less than that in the *H. pylori*-negative group. However, the microbiota interaction in group EFHp.a involved a single species, and the symbiosis was reduced compared with that in group nAGHp.a (**Figure 6C**). The specific parameters of bacteria-bacteria interactions network analysis are shown in **Table S2**.

### Metagenomic function prediction by PICRUSt

**Figure 7:**
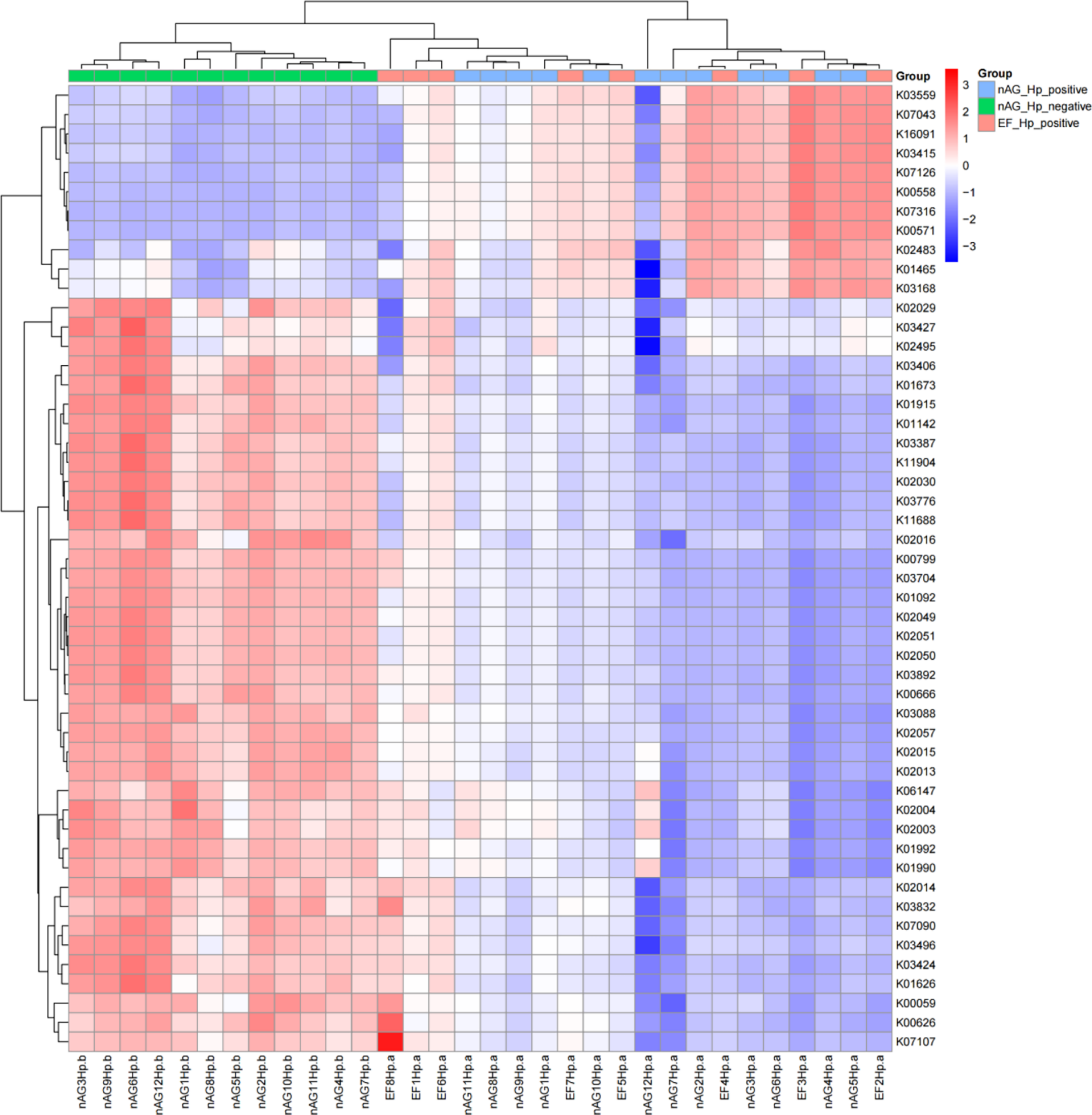
KEGG ontologies heatmap.

**Figure 8:**
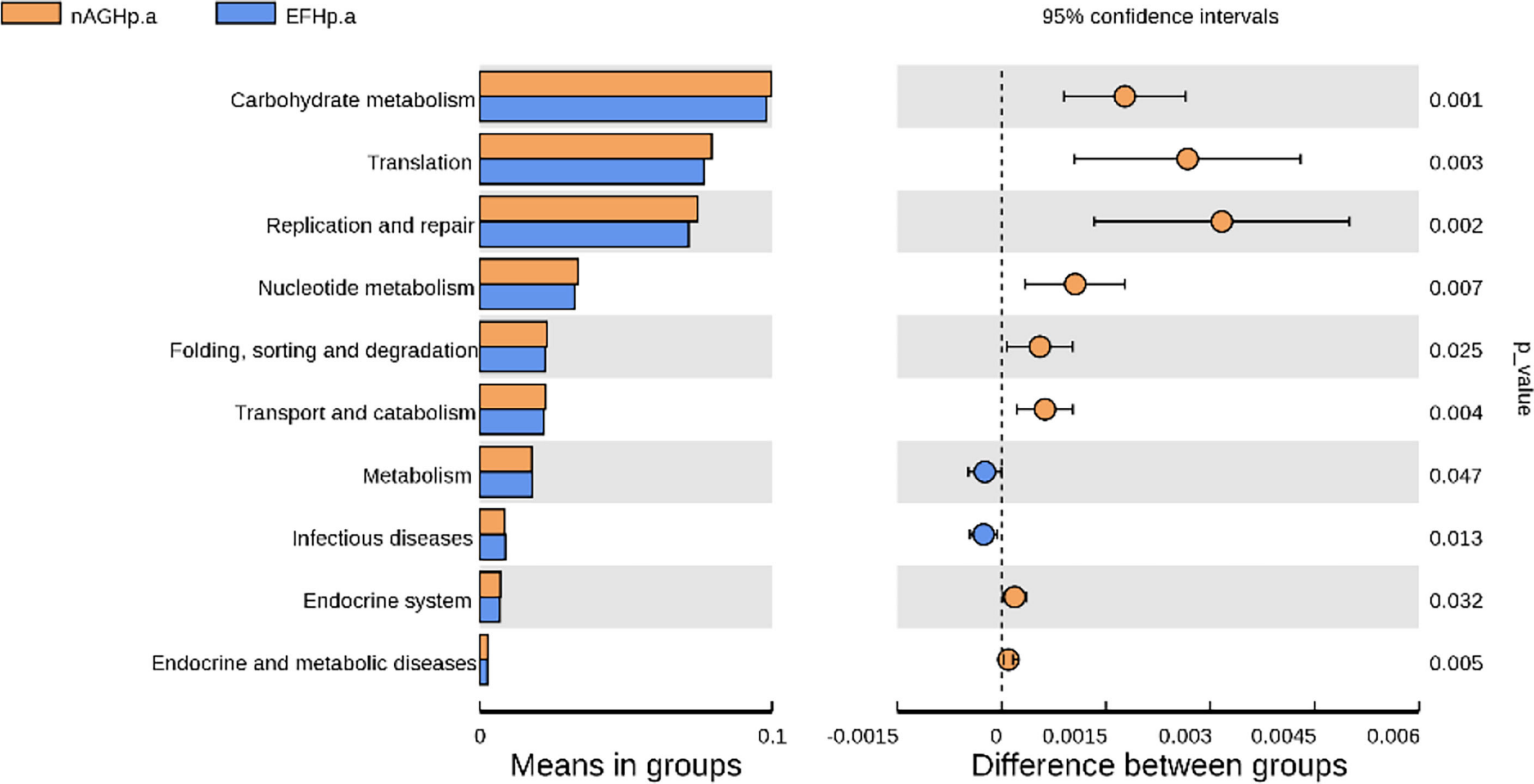
The predicted gastric microbiota function in KEGG pathway at levels 2 between group nAGHp.a and group EFHp.a.

To characterize the metagenomic function alterations in the gastric microbiota between *H. pylori-*infected individuals, healthy people, and refractory *H. pylori-*infected individuals, the relative abundance of KEGG ontologies predicted by PICRUSt was calculated based on the 16S rRNA sequencing data. **Figure 7** shows the heatmap of KEGG ontologies among the three groups. It can be seen from the cluster that the KEGG ontology blocks of the *H. pylori*-negative group and the *H. pylori*-positive group were distributed in different quadrants, and the bacterial gene function was different, but there was an overlap between the nAGHp.a group and the EFHp.a group, so further analysis of KO differences between the two groups was performed. A total of 57 KEGG ontologies were found to differ in abundance between the nAGHp.a group and the EFHp.a group (*P* < 0.05, **Figure S2**) (8 KEGG ontologies enriched in the EFHp.a group and 49 KEGG ontologies depleted in the EFHp.a group). We also found a higher abundance in the pathways of metabolism and infectious disease in group EFHp.a than in group nAGHp.a at level 2 (**Figure 8**). The predicted gastric microbiota functions in the KEGG pathway at level 1 and level 3 between group nAGHp.a and group EFHp.a. are detailed in **Figure S3**. The results of FDR analysis of the KEGG pathway are detailed in **Supplementary Material 1**.

## Discussion

Even though only 1% to 3% of *H. pylori*-infected individuals develop malignant complications, *H. pylori* infections account for 15% of the total cancer burden globally, with up to 89% of all cases of gastric cancer attributable to *H. pylori* infection (Shah et al., 2021). Because of the high antimicrobial resistance, curing *H. pylori* infection is difficult, as the eradication rates are often <70% (Malfertheiner et al., 2017). The treatment of refractory *H. pylori* infection has become a therapeutic challenge in clinical practice (Katelaris and Katelaris, 2017). In recent years, probiotic supplementation, such as the use of *Lactobacillus*, *Bifidobacterium*, and *Saccharomyces boulardii*, has been used to treat refractory *H. pylori* infection (Tong et al., 2007; Ji and Yang, 2020; Viazis et al., 2022). However, the specific mechanism of probiotics is unknown.

The human microbiome is an intrinsic element in the lifelong maintenance of health and immune system homeostasis, and any shift in the microbial composition could have an adverse effect on the human host (Doocey et al., 2022). However, studies of the gastric microbiota in patients with refractory *H. pylori* infection are currently limited. Thus, it is important to determine the diversity, composition, and function of the gastric microbiota in patients with refractory *H. pylori* infection according to the results of 16S rRNA sequencing analysis.

This study explored the gastric microbiota characteristics in patients with refractory *H. pylori* infection based on gastric mucosal samples, which reflects the microecological influence of multiple eradication histories of *H. pylori* on the stomach. *Proteobacteria* were the dominant bacteria in patients in whom eradication of *H. pylori* failed many times. However, *Lactobacillus*, which are known to be beneficial to human health, were decreased in patients with refractory *H. pylori* infection compared with *H. pylori*-positive patients without treatment. In this study, we also found that the richness and diversity of gastric species were lower in *H. pylori*-positive patients than in *H. pylori*-negative patients according to alpha diversity indexes. In recent years, it has been reported that there is a significant difference in the gastric mucosal microbiota between *H. pylori*-positive and *H. pylori*-negative subjects (Murata et al., 2018; Park et al., 2019; Miftahussurur et al., 2020), and that *H. pylori* infection has also been found to have a significant impact on the structure of the gastric microbiota in animal models (He et al., 2016; Kienesberger et al., 2016; Miao et al., 2020). Among individuals infected with *H. pylori*, the dominant bacterial categories in the stomach are mainly *Proteobacteria*, *Firmicutes*, and *Actinobacteria*. *H. pylori* infection could increase the relative abundance of *Proteobacteria*, *Spirochetes*, and *Acidobacteria* and reduce the relative abundance of *Actinobacteria*, *Bacteroidetes*, and *Firmicutes* (Maldonado-Contreras et al., 2011). Our study found that the relative abundance of *Proteobacteria* in the *H. pylori*-negative group was higher than that in the *H. pylori*-positive group, possibly because we classified *Helicobacter* as the other phylum. Indeed, our study found that the relative abundance of *Helicobacter* in the *H. pylori*-positive group was significantly higher than that in the *H. pylori*-negative group at the genus level. Klymiuk et al (Klymiuk et al., 2017)explored the effect of *H. pylori* infection on the gastric microbiota and found that the relative abundances of *Lactobacillus* in *H. pylori*-positive samples were significantly reduced, while the relative abundance of *H. pylori* was significantly increased. Schulz et al (Schulz et al., 2018)detected the microbiota in saliva, gastric mucosa, gastric juice, duodenal mucosa, and duodenal juice in the upper digestive tract by 16S rRNA sequencing, and found that *H. pylori* infection significantly affected the microbiota structure of the gastric mucosa. All of the above research results were consistent with our findings. An Indian study found that the abundance of *H. pylori* was negatively correlated with the diversity of the microbiota. Network analysis showed that the interaction between *H. pylori* and other gastric microorganisms was negatively correlated with intragroup symbiosis/cooperation (Das et al., 2017), which was consistent with our study. However, previous studies did not involve subjects with refractory *H. pylori* infection.

In this study, gastric mucosal samples of eight subjects with multiple eradication histories of *H. pylori* were collected and compared with the gastric mucosal microbiota of first-time positive subjects. Although there was no significant difference in species diversity and the microbiota structure of the refractory *H. pylori*-positive group was similar to that of the *H. pylori*-positive group without treatment, it was found that the relative abundance of beneficial *Lactobacillus* in the stomach of first-time positive subjects was higher than that in the refractory *H. pylori* infection group by LEfSe. This may be related to the negative correlation between *H. pylori* and other gastric bacteria or the use of antibiotics, PPIs, and other *H. pylori* eradication drugs. Previous studies have shown that PPI can significantly change the composition and species diversity of the gastric mucosal microbiota (Amir et al., 2014; Paroni et al., 2016; Corsonello et al., 2018; Shi et al., 2019), resulting in the excessive growth of some bacteria in the stomach. Clarithromycin, amoxicillin, and quinolones have been confirmed to lead to an imbalance in gastrointestinal microbiota (Rashid et al., 2015; Arboleya et al., 2016; Korpela et al., 2016; Iizumi et al., 2017). However, how long it takes to recover the gastric mucosal microbiota remains unclear. This study suggested that we may choose to supplement probiotics in advance to adjust the gastric microecology for *H. pylori* eradication in the future. Previous research has not analyzed bacteria-bacteria interactions in great detail. In our study, we found that the microbiota interactions in the refractory *H. pylori*-positive group involved a single species and that the symbiosis was reduced compared with the *H. pylori*-positive without the treatment group.

Otherwise, the gastric microbiota functions between the refractory *H. pylori*-positive group and the *H. pylori*-positive without treatment group were predicted based on the microbiota abundance and KEGG database in this study. The results revealed a higher abundance in the pathways of metabolism and infectious diseases in the refractory *H. pylori* infection group than in the *H. pylori*-positive group without treatment. The upregulated pathways of infectious diseases were consistent with the clinical pathogenicity of *H. pylori*, which could cause gastric cancer, and we estimated that patients with refractory *H. pylori* infection were at a higher risk of developing gastric cancer than other *H. pylori*-positive patients.

To the best of our knowledge, this was the first comprehensive analysis to identify gastric microbiota diversity, composition, and function in refractory *H. pylori* infection. We collected gastric mucosal samples and drug information from subjects in whom eradication of *H. pylori* failed multiple times. However, this study was limited by the small sample size.

In conclusion, *H. pylori* infection was an important factor affecting the diversity, composition, and function of the gastric microbiota. Significant alterations occurred in the gastric microbiota in patients in whom eradication of *H. pylori* failed multiple times. Multiple *H. pylori* eradication histories lead to an imbalance in the gastric mucosal microbiota to a certain extent, which was mainly reflected by the growth of beneficial *Lactobacillus* being inhibited in the stomach. Patients with refractory *H. pylori* infection may be at a higher risk of developing gastric cancer than other *H. pylori*-positive patients.

## Data availability statement

The datasets presented in this study can be found in online repositories. The names of the repository/repositories and accession number(s) can be found in the article/**Supplementary Material**.

## Ethics statement

This study was approved by the Ethics Committee of the First Affiliated Hospital of Nanchang University (2018-019-1). The patients/participants provided their written informed consent to participate in this study.

## Author contributions

DL and YX designed the experiment and supervised the study; JW contributed to formal analysis and wrote the manuscript; YX and DL reviewed and revised the manuscript. All authors read and approved the final manuscript.

## Funding

This study was supported by the National Natural Science Foundation of China [grant number: 81970502, 81460115,82060109], the National Key Research and Development Program of China [2016YFC1302201], the Key Research and Development Program of Jiangxi Province [20203BBG73051], the Science and Technology Research Project of Jiangxi Education Department [GJJ180047], the Scientific Research Project of the Drug Administration of Jiangxi Province[2020JS22].

## Conflict of interest

The authors declare that the research was conducted in the absence of any commercial or financial relationships that could be construed as a potential conflict of interest.

## Publisher’s note

All claims expressed in this article are solely those of the authors and do not necessarily represent those of their affiliated organizations, or those of the publisher, the editors and the reviewers. Any product that may be evaluated in this article, or claim that may be made by its manufacturer, is not guaranteed or endorsed by the publisher.
