# Supplementary material for: Refractory Helicobacter pylori infection and the gastric microbiota
Source: Front Cell Infect Microbiol. 2022 Sep 27;12:976710. doi: 10.3389/fcimb.2022.976710 (PMC9552320; doi:10.3389/fcimb.2022.976710)
Supplement: Supplementary file 1 [file DataSheet_1.pdf]

Taxa	avg(nAGHp.a)	sd(nAGHp.a)	avg(EFHp.a)	sd(EFHp.a)	p.value	q.values
K06147	0.0057959	0.0006201	0.0051336	0.0002622	0.0046963	0.0376412
K02004	0.0030362	0.0005606	0.0022647	0.0004892	0.0047720	0.0378540
K03046	0.0023507	0.0000725	0.0022508	0.0000694	0.0070910	0.0439864
K03043	0.0022954	0.0000689	0.0021961	0.0000656	0.0051338	0.0395807
K03657	0.0022660	0.0001266	0.0020761	0.0001074	0.0022317	0.0326050
K03701	0.0021423	0.0002102	0.0018958	0.0001089	0.0031388	0.0330842
K02337	0.0019621	0.0000924	0.0018583	0.0000360	0.0030712	0.0328504
K01952	0.0018745	0.0000966	0.0017776	0.0000257	0.0055739	0.0407539
K02355	0.0017427	0.0001252	0.0015797	0.0000807	0.0023409	0.0328011
K01870	0.0016861	0.0000683	0.0015980	0.0000469	0.0030826	0.0328504
K03070	0.0016570	0.0000728	0.0015542	0.0000451	0.0010608	0.0307940
K00615	0.0015959	0.0000550	0.0015388	0.0000313	0.0086546	0.0484317
K01873	0.0015792	0.0000609	0.0015126	0.0000386	0.0078277	0.0464868
K01872	0.0015490	0.0000596	0.0014715	0.0000418	0.0030919	0.0328504
K01869	0.0014986	0.0000580	0.0014306	0.0000391	0.0057365	0.0407539
K01153	0.0015397	0.0001980	0.0013472	0.0000873	0.0090583	0.0494321
K03555	0.0013815	0.0000541	0.0013186	0.0000318	0.0042895	0.0364672
K04043	0.0013155	0.0000711	0.0012181	0.0000649	0.0059934	0.0408110
K01990	0.0013304	0.0001081	0.0011591	0.0000668	0.0003660	0.0301688
K03655	0.0012729	0.0000718	0.0011736	0.0000371	0.0008164	0.0307940
K04066	0.0012214	0.0000422	0.0011627	0.0000290	0.0016809	0.0307940
K13821	0.0011049	0.0000983	0.0012452	0.0000876	0.0040496	0.0362071
K06158	0.0012104	0.0000739	0.0011181	0.0000458	0.0029016	0.0328294
K02003	0.0013599	0.0003295	0.0009830	0.0002246	0.0070357	0.0438939
K01868	0.0011479	0.0000505	0.0010867	0.0000318	0.0037529	0.0356504
K03703	0.0010812	0.0000344	0.0010299	0.0000329	0.0041332	0.0362118
K09687	0.0012589	0.0003178	0.0009138	0.0001717	0.0058474	0.0407539
K06207	0.0010648	0.0000520	0.0010017	0.0000246	0.0021073	0.0325820
K03596	0.0010591	0.0000470	0.0009986	0.0000270	0.0018761	0.0313219
K00982	0.0009463	0.0000940	0.0010622	0.0000602	0.0034896	0.0347209
K03495	0.0010299	0.0000468	0.0009811	0.0000213	0.0059674	0.0407539
K01876	0.0010262	0.0000478	0.0009766	0.0000257	0.0078339	0.0464868
K09686	0.0010476	0.0000766	0.0009572	0.0000481	0.0045733	0.0373034
K01874	0.0010315	0.0000833	0.0009484	0.0000261	0.0060559	0.0408573
K00384	0.0010232	0.0000652	0.0009304	0.0000449	0.0014103	0.0307940

level 1	avg(nAGHp.	sd(nAGHp.a	avg(EFHp.a)	sd(EFHp.a)	p.value
Genetic Information Processing	0.1943272	0.0051995	0.1878848	0.0020707	0.0014701
Human Diseases	0.0325723	0.0006590	0.0333906	0.0005557	0.0082516
Organismal Systems	0.0169749	0.0003252	0.0167304	0.0001338	0.0337954

q.values
0.0163628
0.0459208
0.1253834

level 2	avg(nAGHp.	sd(nAGHp.a
Metabolism;Carbohydrate metabolism	0.0997579	0.0013183
Genetic Information Processing;Translation	0.0793625	0.0020252
Genetic Information Processing;Replication and repair	0.0746433	0.0027114
Metabolism;Nucleotide metabolism	0.0335173	0.0010867
Genetic Information Processing;Folding, sorting and degradation	0.0229085	0.0004906
Cellular Processes;Transport and catabolism	0.0225649	0.0004715
Unclassified;Metabolism	0.0177882	0.0003115
Human Diseases;Infectious diseases	0.0085107	0.0002151
Organismal Systems;Endocrine system	0.0070591	0.0001354
Human Diseases;Endocrine and metabolic diseases	0.0027182	0.0000764

avg(EFHp.a)	sd(EFHp.a)	p.value	q.values
0.0979867	0.0004218	0.0006754	0.0130596
0.0766891	0.0014307	0.0028267	0.0182187
0.0714769	0.0010117	0.0022219	0.0182187
0.0324595	0.0003265	0.0070043	0.0225718
0.0223582	0.0004837	0.0253013	0.0611516
0.0219411	0.0003765	0.0043771	0.0207182
0.0180289	0.0001942	0.0474527	0.0917522
0.0087691	0.0001960	0.0133936	0.0369961
0.0068721	0.0001879	0.0322835	0.0693576
0.0026204	0.0000606	0.0053576	0.0207182

level 3
Genetic Information Processing;Replication and repair;DNA_repair_and_recombination_proteins
Metabolism;Nucleotide metabolism;Purine_metabolism
Genetic Information Processing;Translation;Transfer_RNA_biogenesis
Metabolism;Amino acid metabolism;Amino_acid_related_enzymes
Metabolism;Nucleotide metabolism;Pyrimidine_metabolism
Genetic Information Processing;Translation;Ribosome
Genetic Information Processing;Translation;Aminoacyl-tRNA_biosynthesis
Genetic Information Processing;Translation;Mitochondrial_biogenesis
Metabolism;Carbohydrate metabolism;Glycolysis/Gluconeogenesis
Metabolism;Amino acid metabolism;Alanine,_aspartate_and_glutamate_metabolism
Genetic Information Processing;Replication and repair;DNA_replication_proteins
Unclassified;Metabolism;Energy_metabolism
Metabolism;Amino acid metabolism;Cysteine_and_methionine_metabolism
Metabolism;Carbohydrate metabolism;Amino_sugar_and_nucleotide_sugar_metabolism
Environmental Information Processing;Membrane transport;Bacterial_secretion_system
Genetic Information Processing;Replication and repair;Homologous_recombination
Genetic Information Processing;Replication and repair;Mismatch_repair
Genetic Information Processing;Transcription;Transcription_machinery
Metabolism;Carbohydrate metabolism;Starch_and_sucrose_metabolism
Metabolism;Glycan biosynthesis and metabolism;Peptidoglycan_biosynthesis
Genetic Information Processing;Translation;Translation_factors
Genetic Information Processing;Replication and repair;Nucleotide_excision_repair
Genetic Information Processing;Replication and repair;DNA_replication
Metabolism;Metabolism of other amino acids;Glutathione_metabolism
Cellular Processes;Transport and catabolism;Prokaryotic_Defense_System
Metabolism;Amino acid metabolism;Phenylalanine,_tyrosine_and_tryptophan_biosynthesis
Unclassified;Genetic information processing;Protein_folding_and_associated_processing
Metabolism;Energy metabolism;Sulfur_metabolism
Metabolism;Amino acid metabolism;Lysine_biosynthesis
Metabolism;Metabolism of other amino acids;Selenocompound_metabolism
Metabolism;Carbohydrate metabolism;Galactose_metabolism
Genetic Information Processing;Replication and repair;Base_excision_repair
Unclassified;Cellular processes and signaling;Membrane_and_intracellular_structural_molecules
Metabolism;Metabolism of cofactors and vitamins;Thiamine_metabolism
Metabolism;Lipid metabolism;Glycerolipid_metabolism
Metabolism;Glycan biosynthesis and metabolism;Lipopolysaccharide_biosynthesis
Genetic Information Processing;Transcription;RNA_polymerase
Cellular Processes;Cell motility;Cytoskeleton_proteins
Metabolism;Metabolism of other amino acids;Cyanoamino_acid_metabolism
Genetic Information Processing;Folding, sorting and degradation;Sulfur_relay_system
Human Diseases;Cancers;Central_carbon_metabolism_in_cancer
Metabolism;Energy metabolism;Photosynthesis
Metabolism;Metabolism of cofactors and vitamins;Ubiquinone_and_other_terpenoid-quinone_biosy
Human Diseases;Drug resistance;Vancomycin_resistance
Unclassified;Cellular processes and signaling;Inorganic_ion_transport_and_metabolism
Organismal Systems;Endocrine system;Glucagon_signaling_pathway
Metabolism;Xenobiotics biodegradation and metabolism;Drug_metabolism-other_enzymes
Unclassified;Metabolism;Metabolism_of_cofactors_and_vitamins
Metabolism;Metabolism of other amino acids;D-Glutamine_and_D-glutamate_metabolism
Metabolism;Biosynthesis of other secondary metabolites;Phenylpropanoid_biosynthesis
Human Diseases;Endocrine and metabolic diseases;Insulin_resistance
Metabolism;Lipid metabolism;Sphingolipid_metabolism

avg(nAGH	sd(nAGHp	avg(EFHp.	sd(EFHp.a	p.value	q.values
0.026581	0.000765	0.025543	0.000296	0.000672	0.023161
0.01965	0.000514	0.019152	0.000172	0.007553	0.051889
0.019051	0.000701	0.01839	0.000206	0.008501	0.053257
0.014571	0.000503	0.014077	0.000134	0.006414	0.048642
0.013868	0.000574	0.013308	0.000161	0.006797	0.048642
0.01206	0.000358	0.01157	0.000334	0.006695	0.048642
0.011321	0.000416	0.010802	0.000211	0.001886	0.039724
0.011132	0.000228	0.010753	0.000366	0.024984	0.072728
0.010673	0.000351	0.010261	0.000176	0.00293	0.039724
0.010112	0.000209	0.009946	0.000101	0.030642	0.076272
0.009504	0.000544	0.00902	0.000212	0.013795	0.060751
0.008421	0.000251	0.008609	0.000129	0.042301	0.094038
0.008116	0.000208	0.007896	5.79E-05	0.003932	0.039724
0.007971	0.00056	0.007334	0.000427	0.010064	0.054018
0.007189	0.000204	0.007506	0.000189	0.002599	0.039724
0.006823	0.000364	0.006462	0.000225	0.013335	0.060751
0.006555	0.000377	0.006136	0.000153	0.00343	0.039724
0.006418	0.000176	0.006202	0.000108	0.003239	0.039724
0.006306	0.000695	0.005537	0.00078	0.040631	0.09182
0.005811	0.000248	0.00556	8.28E-05	0.005688	0.048642
0.005597	0.000159	0.005414	0.000161	0.024578	0.072728
0.005334	0.000228	0.005015	0.000112	0.000674	0.023161
0.005253	0.000251	0.004985	7.55E-05	0.003817	0.039724
0.004846	0.000376	0.005201	0.000342	0.043842	0.09439
0.005249	0.000444	0.004861	0.000182	0.01573	0.061403
0.005023	0.000154	0.004906	3.45E-05	0.025773	0.07324
0.004383	9.02E-05	0.004466	2.63E-05	0.009117	0.053257
0.004194	0.000152	0.004423	7.98E-05	0.000375	0.023161
0.004047	0.000191	0.003882	6.33E-05	0.014427	0.061403
0.003842	6.63E-05	0.003763	3.23E-05	0.002479	0.039724
0.003659	0.000598	0.003109	0.000389	0.0228	0.072728
0.003281	7.13E-05	0.003179	3.35E-05	0.000473	0.023161
0.003083	0.000119	0.003264	0.000102	0.002045	0.039724
0.003147	0.000193	0.002988	6.8E-05	0.019653	0.06751
0.002786	8.82E-05	0.002712	3.7E-05	0.019205	0.06751
0.002392	0.000151	0.002517	8.2E-05	0.028541	0.075309
0.002472	8.21E-05	0.002373	5.07E-05	0.003589	0.039724
0.002145	6.72E-05	0.002088	4.21E-05	0.033239	0.081554
0.001903	8.75E-05	0.001812	6.1E-05	0.013461	0.060751
0.001859	4.37E-05	0.001801	3.82E-05	0.006255	0.048642
0.001728	4.36E-05	0.001678	3.99E-05	0.016457	0.062811
0.001648	5.76E-05	0.001589	5.52E-05	0.035821	0.084922
0.001569	0.000102	0.001659	6.98E-05	0.030533	0.076272
0.001636	6.85E-05	0.001572	1.84E-05	0.008746	0.053257
0.001525	9.44E-05	0.001622	4.26E-05	0.006501	0.048642
0.001634	0.000124	0.001497	9.1E-05	0.01084	0.054154
0.001595	9.61E-05	0.001501	4.15E-05	0.008625	0.053257
0.00119	5.1E-05	0.00125	4.25E-05	0.01169	0.05577
0.001101	1.93E-05	0.001073	1.49E-05	0.001888	0.039724
0.00105	0.000102	0.000968	4.75E-05	0.02894	0.075309
0.001051	0.00011	0.000931	0.000101	0.023448	0.072728
0.001009	0.000226	0.000822	8.32E-05	0.019497	0.06751
