# Supplementary material for: Refractory Helicobacter pylori infection and the gastric microbiota
Source: Front Cell Infect Microbiol. 2022 Sep 27;12:976710. doi: 10.3389/fcimb.2022.976710 (PMC9552320; doi:10.3389/fcimb.2022.976710)
Supplement: Supplementary file 2 [file Image_1.pdf]

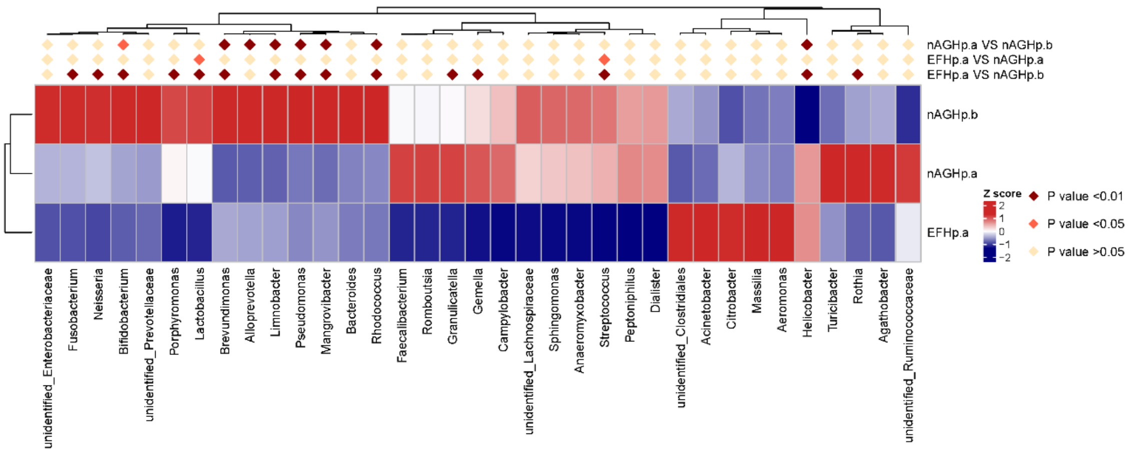

Figure S1. Heatmap of different species among three groups.

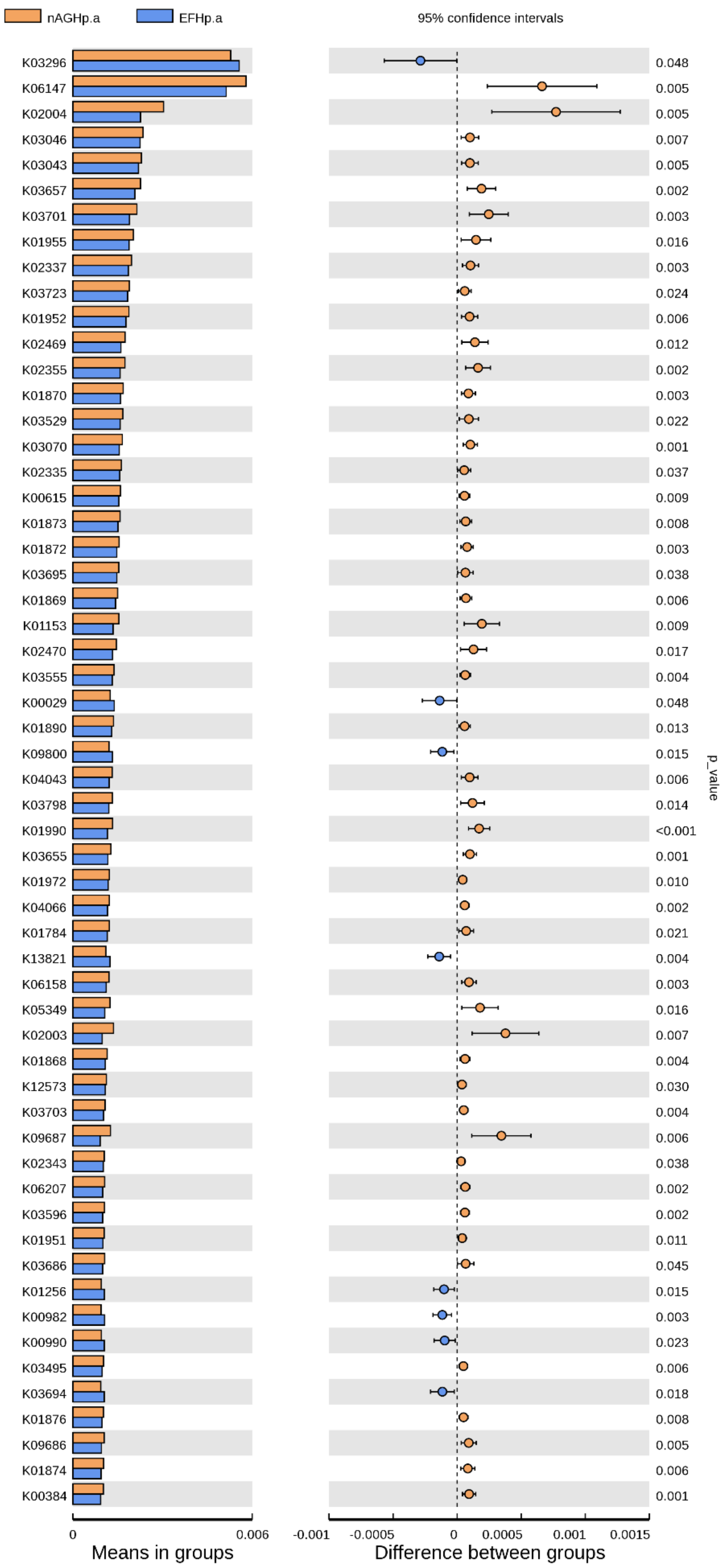

Figure S2. The predicted gastric microbiota function in KEGG ontologies between group nAGHp.a and group EFHp.a.

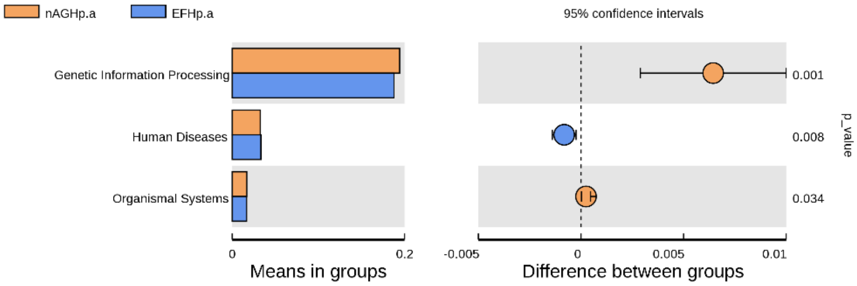

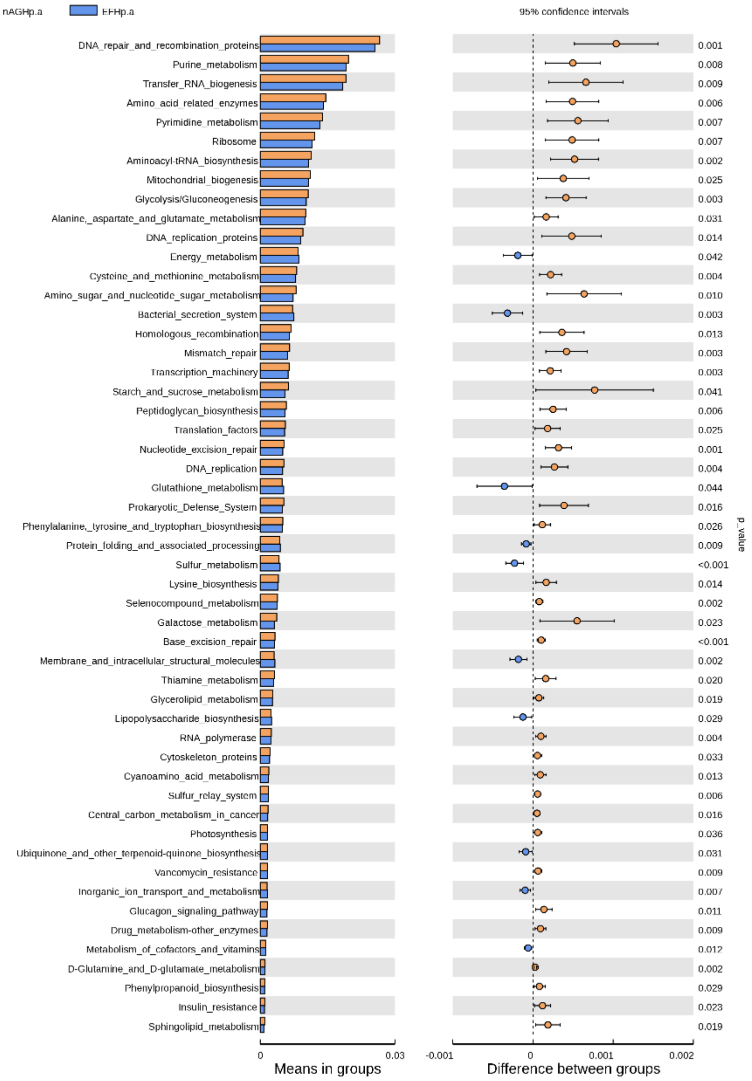

Figure S3. The predicted gastric microbiota function in KEGG pathway at level 1 and level 3 between group nAGHp.a and group EFHp.a.
