# Supplementary material for: Refractory Helicobacter pylori infection and the gastric microbiota
Source: Front Cell Infect Microbiol. 2022 Sep 27;12:976710. doi: 10.3389/fcimb.2022.976710 (PMC9552320; doi:10.3389/fcimb.2022.976710)
Supplement: Supplementary file 3 [file Table_1.docx]

Table S1. The specific information of 8 patients with refractory *H. pylori* infection.

| Sample ID | Eradication times | First time | Second time | Third time |
| --- | --- | --- | --- | --- |
| EFHp.a1  EFHp.a2  EFHp.a3  EFHp.a4  EFHp.a5  EFHp.a6 | 3  2  3  3  3  2 | PBAF10d  PBAF14d  PBAF10d  PBAF14d  PBAF10d PBAF14d | PBAC10d  PBAC14d  -  -  PCA10d  PBAF14d | PBAL10d  -  -  - |
| EFHp.a7 | 2 | PBAF10d | - |  |
| EFHp.a8 | 2 | PBAF10d | PBAF14d |  |

“-”: absence;“P”: proton-pump inhibitors; “B”: bismuth;“A”: amoxicillin;“F”: furazolidone;“C”: clarithromycin;“L”: levofloxacin.

Table S2. Specific parameters of network analysis.

|  | ND | MD | CC | GD | AD | APL |
| --- | --- | --- | --- | --- | --- | --- |
| nAGHp.b | 12 | 0.5139828 | 0.4958189 | 0.04696517 | 18.88 | 3.884955 |
| nAGHp.a | 11 | 0.4335873 | 0.5090401 | 0.08121891 | 32.65 | 3.011349 |
| EFHp.a | 9 | 0.3926066 | 0.5701374 | 0.07824121 | 31.29648 | 3.243247 |

Note: ND (Network diameter), the maximum measurement length of the network diameter, that is, any two points have a shortest distance, and the maximum value of these shortest distances is the network diameter ; average degree (AD), the average connection degree, namely the number of edges connected by the node, is the sum of connection degrees of all nodes divided by the total number of nodes ; MD (modularity), modularity and network graph modularity measure the modularity of the network community structure, and describe the rationality of the network being divided into different modules, or the distinction between different modules ; CC (clustering coefficient), is the possibility of adjacent nodes connected to each other, the connectivity of the network graph is the average connectivity of all nodes; GD ( graph density ), network graph density, actual number of edges divided by all possible number of edges ; APL ( average. path. length/mean _distance), there is a shortest path between each pair of nodes, and the average path length is the sum of the shortest distances of all nodes divided by the number of nodes.
